# Dataset on thermodynamics performance analysis and optimization of a reheat – regenerative steam turbine power plant with feed water heaters

**DOI:** 10.1016/j.dib.2020.106086

**Published:** 2020-07-25

**Authors:** S.O. Oyedepo, O. Kilanko, M.A. Waheed, O.S.I. Fayomi, O.S. Ohunakin, P.O. Babalola, S.O. Ongbali, C.N. Nwaokocha, B. Mabinuori, O.O. Shopeju

**Affiliations:** a Department of Mechanical Engineering, Covenant University, Nigeria; b Department of Mechanical Engineering, Federal University of Agriculture, Abeokuta, Nigeria; c The Energy and Environment Research Group (TEERG), Mechanical Engineering Department, Covenant University, Ogun State, Nigeria; d Senior Research Associate, Faculty of Engineering & the Built Environment, University of Johannesburg, South Africa; e Department of Mechanical Engineering, Olabisi Onabanjo University, Ibogun Campus, Nigeria

**Keywords:** Power plant performance, Feedwater heaters (FWHs), Cycle efficiency, Reheat-regenerative cycle, Specific fuel consumption

## Abstract

Steam power plants have a considerable potential to meet the growing energy demand, but its dependence on conventional fossil fuels has hampered its viability. One of the ways to minimize fuel consumption and upgrade the performance of a Rankine cycle is by incorporating closed feedwater heaters(FWHs). The datasets contained in this paper are thermodynamic performance analysis carried out on reheat – regenerative steam power plant with FWHs using CyclePad V2.0 software. The thermodynamic performance indices assessed are thermal efficiency, network output, heat rate, fuel consumption, boiler efficiency and specific steam consumption. Result obtained show that an increase in the number of FWHs decreases the fuel consumption, heat rate, heat rejected in condenser and heat input to the cycle. This effect invariably can lead to a reduction in operating cost and environmental impacts.

Specifications Table

**Table:** 

Subject	Mechanical Engineering
Specific Subject area	Thermodynamics and Thermal Engineering
Type of Data	Tables, Figures and graphs
How Data Were Acquired	Technical data sheet, analysed using software and processed output data.
Data Format	Secondary and analysed
Parameters for Data Collection	Temperature, pressure, enthalpy, power output, specific fuel consumption, heat rate and thermal efficiency inputs/outputs
Description of Data Collection	Inputs temperature and pressure and design parameters of the selected steam power plant were obtained from the efficiency department, Egbin Steam Power Plant, Lagos, Nigeria.
Data source location	Efficiency Department, Egbin Steam Power Plant, Lagos, Nigeria
Data Accessibility	Data are available within this article
Related Research Article	Oyedepo, S.O, Fakeye B. A, Mabinuori, B, Babalola, P.O, Leramo, R.O, Kilanko, O, Dirisu J, O, Udo, M, Efemwenkiekie U. K, and Oyebanji J.A, Thermodynamics Analysis And Performance Optimization of a Reheat – Regenerative Steam Turbine Power Plant with Feed Water Heaters, Fuel, 280:118,577

## Value of the Data

• The thermodynamics model data of regenerative- reheat steam power plant can be used to predict the performance indices (thermal efficiency, heat rate, specific fuel consumption, specific steam consumption etc.) of a steam power plant.
• The dataset helps power plant Engineer to predict the maximum number of FWHs required for optimum performance of a steam power plant.
• The dataset in this study can be used to optimize the performance of a regenerative – reheat steam power plant.
• This dataset with other set can be used to assess the exergetic efficiency of regenerative – reheat steam power plant.
• We assessed the effect of variation in the number of closed FWH on the performance indices of steam power plant.
• The data reveals that as the number of FWHs increases, so is the total temperature rise of feed water (**Δ*t***_***fw***_). Hence, by regeneration, less becomes the heat added to water in the boiler, more becomes the mean temperature of heat addition, and more is the cycle efficiency.

## 1 Data description

The thermodynamic properties of steam, including pressure, temperature, enthalpy, entropy, specific volume and mass flow rates at state points of reheat – regenerative cycle (Summary of the operating parameters can be found in [1]) were calculated using cyclepad V2.0 and presented in Table 1, Table 2, Table 3, Table 4, Table 5 for Rankine cycle without FWH, with 1, 2, 3, and 4 FWHs, respectively. The layout of the reheat – regenerative cycle with 4-FWHs generated using cyclepad as a sample can be found in [1].

**Table 1:** State thermodynamic properties for Rankine cycle without FWH.

Location	T(°C)	P(kPa)	h(kJ/kg)	s(kJ/kgK)	V$\left(m^{3}\;/\;kg\right)$	$\dot{\text{m}}\left(\text{kg/s}\right)$
S1	545	12,990	3457	6.59	0.0267	110.6
S2	538	12,500	3444	6.59	0.0275	110.6
S3	541	12,500	3452	6.60	0.0276	110.6
S4	538	12,295	3446	6.60	0.0280	110.6
S5	42.67	8.500	2071	6.60	13.490	110.6
S6	42.67	8.500	2071	6.60	13.490	110.6
S7	43.08	12,990	191.8	0.6078	0.0010	110.6

**Table 2:** State thermodynamic properties for the reheat-regenerative cycle with one FWHs.

Location	T(°C)	P(kPa)	h(kJ/kg)	s(kJ/kgK)	v$\left(m^{3}\;/\;kg\right)$	$\dot{\text{m}}\left(\text{kg/s}\right)$
S1	545	12,990	3457	6.59	0.0267	110.6
S2	538	12,500	3444	6.59	0.0275	110.6
S3	541	12,500	3452	6.60	0.0276	110.6
S4	538	12,295	3446	6.60	0.0280	110.6
S5	538	12,295	3446	6.60	0.0280	108.8
S6	42.67	8.500	2071	6.60	13.49	108.8
S7	42.67	8.500	178.7	0.6078	0.0010	108.8
S8	43.06	12,295	191.1	0.6078	0.0010	108.8
S9	538.0	12,295	3446	6.60	0.0280	1.76
S10	55.53	12,295	242.9	0.7685	0.0010	110.6
S11	55.56	12,990	243.6	0.7685	0.0010	110.6

**Table 3:** State thermodynamic properties for the reheat-regenerative cycle with two FWHs.

Location	T(°C)	P(kPa)	h(kJ/kg)	s(kJ/kgK)	v$\left(m^{3}\;/\;kg\right)$	$\dot{\text{m}}\left(\text{kg/s}\right)$
S1	545.0	12,990	3457	6.59	0.0267	110.6
S2	538.0	12,500	3444	6.59	0.0275	110.6
S3	538.0	12,500	3444	6.59	0.0275	100.6
S4	541.0	12,500	3452	6.60	0.0276	100.6
S5	538.0	12,295	3444	6.60	0.0280	100.6
S6	538.0	12,295	3446	6.60	0.0280	98.83
S7	42.67	8.500	2017	6.60	13.490	98.83
S8	42.67	8.500	178.7	0.6078	0.0010	98.83
S9	43.06	12,295	191.1	0.6078	0.0010	98.83
S10	538.0	12,500	3444	6.59	0.0275	10.01
S11	538.0	12,295	3446	6.60	0.0280	1.760
S12	56.77	12,295	248.0	0.7848	0,0010	100.6
S13	56.78	12,500	248.2	0.7841	0.0010	100.6
S14	125.9	12,500	537.4	1.5800	0.0011	110.6
S15	126.0	12,990	538.0	1.5800	0.0011	110.6

**Table 4:** State thermodynamic properties for reheat- regenerative cycle with three FWHs.

Location	T(°C)	P(kPa)	h(kJ/kg)	s(kJ/kgK)	v($m^{3}\;/\;kg)$	$\dot{\text{m}}\left(\text{kg/s}\right)$
S1	545	12,990	3457	6.5900	0.0267	110.6
S2	538	12,500	3444	6.5900	0.0275	110.6
S3	538	12,500	3444	6.5900	0.0273	94.35
S4	541	12,500	3452	6.6000	0.0276	94.35
S5	538	12,295	3446	6.6000	0.0280	94.35
S6	538	12,295	3446	6.6000	0.0280	92.46
S7	538	12,295	3446	6.6000	0.0280	87.35
S8	42.67	8.50	2071	6.6000	13.490	87.35
S9	42.67	8.50	178.7	0.6078	0.0010	87.35
S10	538	12,500	34,444	6.5900	0.0275	16.22
S11	538	12,295	3446	6.6000	0.0280	1.89
S12	538	12,295	3446	6.6000	0.0280	5.11
S13	43.06	12,295	191.1	0.6078	0.0010	87.35
S14	86.30	12,295	371.0	1.1400	0.0010	92.46
S15	86.30	12,295	371.0	1.1400	0.0010	92.46
S16	101.00	12,295	432.4	1.3100	0.0010	94.35
S17	101.00	12,500	432.7	1.3100	0.0010	94.35
S18	203.90	12,500	874.4	2.3500	0.0012	110.6
S19	204.00	12,990	875.0	2.3500	0.0012	110.6

**Table 5:** State thermodynamic properties for the reheat-regenerative cycle with four FWHs.

Location	T( °C)	P(kPa)	h(kJ/kg)	s(kJ/kgK)	v$\left(m^{3}\;/\;kg\right)$	$\dot{\text{m}}\left(\text{kg/s}\right)$
S1	545	12,990	3457	6.59	0.0267	110.6
S2	538	12,500	3444	6.59	0.0275	110.6
S3	538	12,500	3444	6.59	0.0275	95.59
S4	538	12,500	3444	6.59	0.0275	84.48
S5	541	12,500	3452	6.60	0.0276	84.48
S6	538	12,290	3446	6.60	0.0280	84.48
S7	538	12,295	3446	6.60	0.0280	79.19
S8	538	12,295	3446	6.60	0.0280	75.19
S9	42.67	8.500	2071	6.60	13.49	75.19
S10	42.67	8.500	178.7	0.6874	0.0010	75.19
S11	43.06	12,295	191.1	0.6078	0.0010	75.19
S12	538	12,500	3444	6.59	0.0275	15.01
S13	538	12,500	3444	6.69	0.0275	11.12
S14	538	12,295	3444	6.60	0.0280	5.32
S15	538	12,295	3446	6.60	0.0280	3.97
S16	82.30	12,295	354.3	1.09	0.0010	79.16
S17	82.30	12,295	354.3	1.09	0.0010	79.16
S18	128.7	12,295	548	1.61	0.0011	84.48
S19	128.7	12,500	549.1	1.61	0.0011	84.48
S20	206.4	12,500	885.7	2.37	0.0012	95.59
S21	206.4	12,500	885.7	2.37	0.0012	95.59
S22	279.9	12,500	1233	3.05	0.0013	110.6
S23	280.1	12,990	1233	3.05	0.0013	110.6

**Figure 1:**
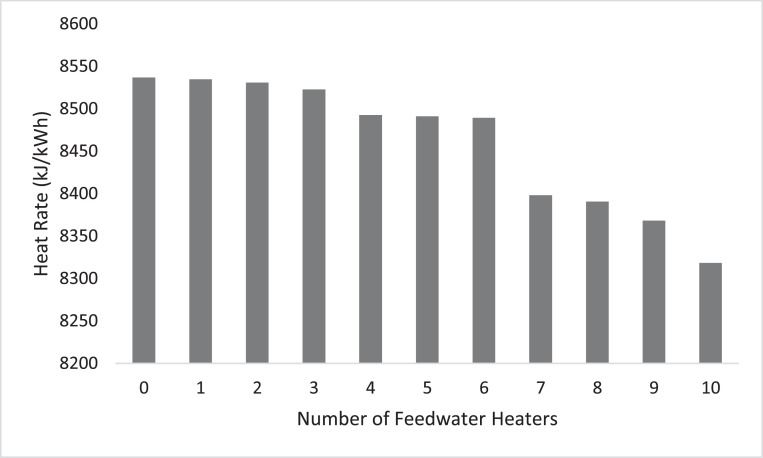
Plot of heat rate against number of FWHs.

To assess the optimal performance of FWH, the number of FWHs was increased from 5 to 10, and the performance parameter – heat rate is presented in Fig. 1. Fig. 1 compares the computed heat rate (using CyclePad) for the power cycle without FWH and with one to ten FWHs.

**Figure 2:**
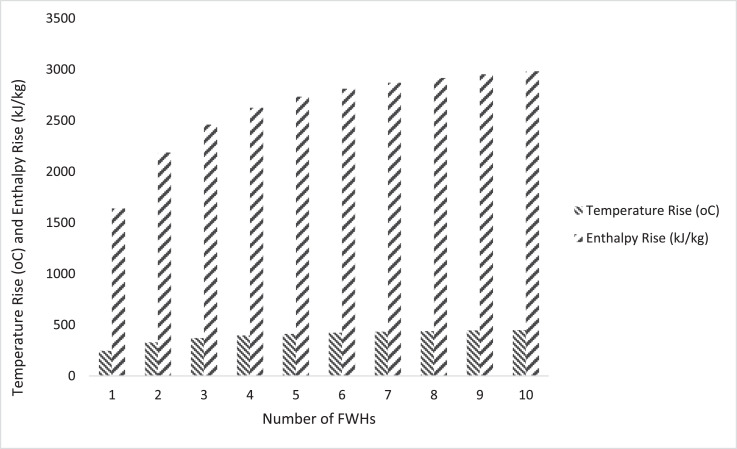
Plot of total enthalpy rise and total temperature rise of reheat -regeneration cycle with FWHs.

The total enthalpy rise and total temperature rise of FWH in the reheat - regenerative cycle were computed and presented in Fig. 2. Fig. 2 shows the total enthalpy rise and total temperature rise for the cycle without FWH and cycle with 1 to 10 closed FWHs

## 2 Experimental design, materials and methods

The primary data (plant's operating parameters) were collected from efficiency department of Egbin Steam Power Plant. Egbin power plant consists of 6 units of 220 (6 × 220 MW) reheat – regenerative cycle. It is dual fired (gas and heavy oil) system with modern control equipment, single reheat and six stages regenerative feedwater heating [2, 3]. The design basis of each unit is a nominal 220 MW Reheat- Regenerative cycle [4]

Thermodynamic performance analysis and optimization of the selected reheat – regenerative steam power plant were carried out using cyclepad. Rankine cycle was modified using CyclePad, and the effects of regeneration on the cycle's thermal efficiency, specific fuel consumption, boiler efficiency, specific steam consumption and heat rate were examined.

CyclePad is an articulate virtual laboratory software for analysis of thermodynamic cycles consisting of a collection of components which either takes in heat energy and produces work energy, or takes in work energy and produces transfer of heat energy. And these components are connected together in some appropriate fashion. The components CyclePad recognises include compressors, turbines, heaters, coolers, pumps, mixers, splitters, throttles, and heat exchangers. CyclePad describes connections in terms of the properties of the working fluid flowing between the components. CyclePad performs steady-state analyses of both open and closed cycles. It works in two phases i.e. build mode and analysis mode. In the first phase (build mode), user uses a graphical editor to place components and connect them with stuffs. While in the analysis mode, user specifies the working fluid in use, the modeling assumptions that have to be made and numerical values for the properties of components. As soon as CyclePad receives these information, it draws conclusions based on the design. This includes the structure of the thermodynamic cycle design, in terms of the physical parts and processes of the cycle and how they are connected to one another; plots the T-s and P-v diagrams of the cycle to understand where the working fluid is in its property space. CyclePad is being considered in this dataset work due to its unique features: easy calculation, facilitate sensitivity, ability to detect physically impossible design and to provide numerical reasoning and interface interaction for the users [5].

In this data, the operation of steam power plant is considered in the steady-state condition. The pressure loss throughout the pipelines is assumed negligible. Applying the steady flow energy equation to each of the processes on the basis of a unit mass of fluid, and neglecting changes in kinetic and potential energy, the work and heat quantities are evaluated in terms of the properties of the fluid. Detailed energy analysis of each component to determine the reheat – regenerative steam turbine power plant's performance indices (i.e. cycle efficiency, network output, heat rate, specific steam consumption, specific fuel consumption) using cyclePad software can be found in [1].

## Ethic statement

This study was carried out in accordance with the U.K Guidelines

## Declaration of Competing Interest

The authors declare that they have no known competing financial interests or personal relationships which have, or could be perceived to have, influenced the work reported in this article.

## Appendix Supplementary materials

Image, application 1
