# Supplementary material for: Dataset on thermodynamics performance analysis and optimization of a reheat – regenerative steam turbine power plant with feed water heaters
Source: Data Brief. 2020 Jul 25;32:106086. doi: 10.1016/j.dib.2020.106086 (PMC7397695; doi:10.1016/j.dib.2020.106086)
Supplement: Supplementary file 1 [file mmc1.pdf]

```
;;; Mode: -*- Lisp -*-

;;; CyclePad Diagram File: C:\qrg\CyclePad\Design Library\regen\regen.dsn
;;; Saved at 17:16 on Thursday 05/20/99

(CPAD::DESIGN
  :window-state :MAXIMIZED
  :saved-once? t
  :title "Rankine Cycle with Regeneration"
  :full-file-name #P"C:\\qrg\\CyclePad\\Design Library\\regen\\regen.dsn")

(CPAD::FLOW-TYPE :STEADY)
(CPAD::CYCLE-TYPE :HEAT-ENGINE)


(CPAD::DEVICE
  :type CPAD::PUMP
  :location (168/613 199/891)
  :symbol DATA::PMP2
  :label "PMP2"
  :caption-offset-x 3
  :caption-offset-y 28
  :orientation (0 . :NONE))

(CPAD::DEVICE
  :type CPAD::TURBINE
  :location (331/1226 62/891)
  :symbol DATA::TUR2
  :label "TUR2"
  :caption-offset-x 4
  :caption-offset-y 25
  :orientation (0 . :NONE))

(CPAD::DEVICE
  :type CPAD::MIXER
  :location (124/613 199/891)
  :symbol DATA::MXR1
  :label "MXR1"
  :caption-offset-x 3
  :caption-offset-y 25
  :orientation (0 . :NONE))

(CPAD::DEVICE
  :type CPAD::SPLITTER
  :location (124/613 62/891)
  :symbol DATA::SPL1
  :label "SPL1"
  :caption-offset-x 1
  :caption-offset-y -15
  :orientation (0 . :NONE))

(CPAD::DEVICE
  :type CPAD::HEATER
  :location (40/613 119/891)
  :symbol DATA::HTR1
  :label "HTR1"
  :caption-offset-x 32
  :caption-offset-y 26
  :orientation (0 . :NONE))

(CPAD::DEVICE
  :type CPAD::TURBINE
  :location (80/613 62/891)
  :symbol DATA::TUR1
  :label "TUR1"
  :caption-offset-x 0
  :caption-offset-y 25
  :orientation (0 . :NONE))

(CPAD::DEVICE
  :type CPAD::COOLER
  :location (192/613 130/891)
  :symbol DATA::CLR1
  :label "CLR1"
  :caption-offset-x -29
  :caption-offset-y 1
  :orientation (0 . :NONE))

(CPAD::DEVICE
  :type CPAD::PUMP
```

```
    :location (163/1226 199/891)
    :symbol DATA::PMP1
    :label "PMP1"
    :caption-offset-x 3
    :caption-offset-y 26
    :orientation (0 . :NONE))

  (CPAD::STUFF
    :connector ((70/613 . 199/891) (40/613 . 199/891) (40/613 . 142/891))
    :incpdev-name DATA::PMP1
    :outcpdev-name  DATA::HTR1
    :inport (:NORMAL :out DATA::PMP1)
    :outport (:NORMAL :in DATA::HTR1)
    :caption-offset-x 4
    :caption-offset-y 25
    :symbol DATA::S12
    :label "S7")


  (CPAD::STUFF
    :connector ((113/613 . 199/891) (185/1226 . 199/891))
    :incpdev-name DATA::MXR1
    :outcpdev-name  DATA::PMP1
    :inport (:NORMAL :out DATA::MXR1)
    :outport (:NORMAL :in DATA::PMP1)
    :caption-offset-x 0
    :caption-offset-y 24
    :symbol DATA::S11
    :label "S6")


  (CPAD::STUFF
    :connector ((157/613 . 199/891) (275/1226 . 199/891) (275/1226 . 199/891) (135/613 . 199/891))
    :incpdev-name DATA::PMP2
    :outcpdev-name  DATA::MXR1
    :inport (:NORMAL :out DATA::PMP2)
    :outport (:STRAIGHT :in DATA::MXR1)
    :caption-offset-x 0
    :caption-offset-y 22
    :symbol DATA::S10
    :label "S5")


  (CPAD::STUFF
    :connector ((192/613 . 151/891) (192/613 . 199/891) (179/613 . 199/891))
    :incpdev-name DATA::CLR1
    :outcpdev-name  DATA::PMP2
    :inport (:NORMAL :out DATA::CLR1)
    :outport (:NORMAL :in DATA::PMP2)
    :caption-offset-x 15
    :caption-offset-y 27
    :symbol DATA::S9
    :label "S4")


  (CPAD::STUFF
    :connector ((353/1226 . 62/891) (192/613 . 62/891) (192/613 . 4/33))
    :incpdev-name DATA::TUR2
    :outcpdev-name  DATA::CLR1
    :inport (:NORMAL :out DATA::TUR2)
    :outport (:NORMAL :in DATA::CLR1)
    :caption-offset-x 22
    :caption-offset-y 7
    :symbol DATA::S8
    :label "S3")


  (CPAD::STUFF
    :connector ((135/613 . 62/891) (309/1226 . 62/891))
    :incpdev-name DATA::SPL1
    :outcpdev-name  DATA::TUR2
    :inport (:STRAIGHT :out DATA::SPL1)
    :outport (:NORMAL :in DATA::TUR2)
    :caption-offset-x 1
    :caption-offset-y 22
    :symbol DATA::S7
    :label "S2a")


  (CPAD::STUFF
    :connector ((124/613 . 85/891) (124/613 . 16/81))
```

```
  :incpdev-name DATA::SPL1
  :outcpdev-name  DATA::MXR1
  :inport (:ANGLED :out DATA::SPL1)
  :outport (:ANGLED :in DATA::MXR1)
  :caption-offset-x 24
  :caption-offset-y 3
  :symbol DATA::S6
  :label "S2b")


(CPAD::STUFF
  :connector ((91/613 . 62/891) (113/613 . 62/891))
  :incpdev-name DATA::TUR1
  :outcpdev-name  DATA::SPL1
  :inport (:NORMAL :out DATA::TUR1)
  :outport (:NORMAL :in DATA::SPL1)
  :caption-offset-x 0
  :caption-offset-y 20
  :symbol DATA::S5
  :label "S2")


(CPAD::STUFF
  :connector ((40/613 . 32/297) (40/613 . 62/891) (137/1226 . 62/891))
  :incpdev-name DATA::HTR1
  :outcpdev-name  DATA::TUR1
  :inport (:NORMAL :out DATA::HTR1)
  :outport (:NORMAL :in DATA::TUR1)
  :caption-offset-x 11
  :caption-offset-y 17
  :symbol DATA::S1
  :label "S1")


(CPAD::UNITS
  (TIME :YEARS)
  (SPECIFIC-COST :DOLLARS/KG)
  (FUNDS-FLOW-RATE :DOLLARS/DAY)
  (MONEY :DOLLARS)
  (HEAT-FLUX-PER-DEGREE :W/M^2K)
  (UNIVERSAL-R :KJ/KMOL-K)
  (SPECIFIC-ENTROPY :KJ/KGK)
  (ENTROPY-RATE :KW/K)
  (ENTROPY :KJ/K)
  (REFRIGERATION-RATE :TON)
  (WORK-TRANSFER-RATE :KW)
  (HEAT-TRANSFER-RATE :KW)
  (ENERGY-TRANSFER-RATE :KW)
  (SPECIFIC-ENERGY :KJ/KG)
  (ENERGY :KJ)
  (VELOCITY :M/SEC)
  (AREA :M^2)
  (LENGTH :M)
  (SPECIFIC-VOLUME :M^3/KG)
  (VOLUME-FLOW :M^3/SEC)
  (VOLUME :M^3)
  (DENSITY :KG/M^3)
  (MOLAR-MASS :KG/KMOL)
  (MOLES :KMOL)
  (MASS-FLOW :KG/S)
  (MASS :KG)
  (PRESSURE :KPA)
  (TEMPERATURE :C)
  )

(CPAD::UNITS-MODE :USER)

(CPAD::LOCALE :US)

(CPAD::SHOW-LEGEND NIL)

(CPAD::AUTHOR "Mike Brokowski")

(CPAD::INTERNET
 ("relay.acns.nwu.edu"
  "robota@cs.nwu.edu"
  "brokowski@nwu.edu"))


(CPAD::HISTORY
  (3136227362 3136216813)
```

```
  (3133634488 3133631714)
  (3133630754 3133629583)
  (3091141903 3091141429)
  (3091141406 3091140837)
  (3091138939 3091138645))

(CPAD::COMMENTS)

(CPAD::METER-POSNS
  (CL-USER::S6 (543 423 813 805) (499 68 769 450))
  (CL-USER::S7 (348 107 618 489) (858 43 1128 425))
  (CL-USER::S11 (471 492 741 881) (67 276 337 665))
  (CL-USER::S12 (282 212 552 397) (282 212 552 397))
  (CL-USER::S10 (288 294 558 667) (288 294 558 667))
  (CL-USER::S9 (365 279 635 464) (365 279 635 464))
  (CL-USER::S8 (282 279 551 602) (282 279 552 464))
  (CL-USER::S5 (188 283 458 677) (260 139 530 533))
  (CL-USER::PMP2 (252 257 552 489) (252 257 552 489))
  (CL-USER::TUR2 (348 180 648 412) (348 180 648 412))
  (CL-USER::SPL1 (129 241 429 334) (129 241 429 334))
  (:CYCLE (826 118 1101 408) (826 118 1101 408))
  (CL-USER::S1 (262 376 532 561) (262 376 532 561))
  (CL-USER::S2 (282 289 552 474) (282 289 552 474))
  (CL-USER::TUR1 (322 201 622 433) (322 201 622 433))
  (CL-USER::CLR1 (335 175 635 407) (335 175 635 407))
  (CL-USER::S3 (378 227 648 412) (378 227 648 412))
  (CL-USER::PMP1 (252 257 552 489) (252 257 552 489))
  (CL-USER::S4 (276 238 546 423) (276 238 546 423))
  (CL-USER::HTR1 (279 244 552 464) (279 244 552 464))
  (CL-USER::MXR1 (472 136 772 368) (472 136 772 368)))

(CPAD::ASNS
  (CL-USER::ISOBARIC CL-USER::MXR1)
  (SATURATED CL-USER::S11)
  (== (CL-USER::P CL-USER::S10) (CL-USER::P CL-USER::S5))
  (SATURATED CL-USER::S9)
  (CL-USER::ISENTROPIC CL-USER::PMP2)
  (CL-USER::ADIABATIC CL-USER::PMP2)
  (CL-USER::ADIABATIC CL-USER::TUR2)
  (CL-USER::ISENTROPIC CL-USER::TUR2)
  (SUBSTANCE-OF CL-USER::S1 WATER)
  (CL-USER::ISENTROPIC CL-USER::TUR1)
  (CL-USER::ADIABATIC CL-USER::TUR1)
  (CL-USER::ISOBARIC CL-USER::CLR1)
  (SATURATED CL-USER::S3)
  (CL-USER::ISENTROPIC CL-USER::PMP1)
  (CL-USER::ADIABATIC CL-USER::PMP1)
  (CL-USER::ISOBARIC CL-USER::HTR1)
  (:NOT (CL-USER::CONTROL-FLAG :ISENTROPIC-MEANS-IDEAL))
  (:NOT (CL-USER::CONTROL-FLAG :CONSIDER-VELOCITY))
  (:NOT (CL-USER::CONTROL-FLAG :CONSIDER-ECONOMICS))
  (:NOT (CL-USER::CONTROL-FLAG :USE-AIR-STANDARD))
  (:NOT (CL-USER::CONTROL-FLAG :USE-TENV))
  (:NOT (CL-USER::CONTROL-FLAG :PROPAGATE-SENSITIVITY))
  )

(CPAD::NVALS
  ((CL-USER::DRYNESS CL-USER::S7) 1)
  ((CL-USER::MASS-FLOW CL-USER::S1) 1)
  ((CL-USER::DRYNESS CL-USER::S11) 0)
  ((CL-USER::DRYNESS CL-USER::S9) 0)
  ((CL-USER::P CL-USER::S8) 10000.0)
  ((CL-USER::P CL-USER::S1) 5000000.0)
  ((T CL-USER::S1) 673.15)
  ((CL-USER::P CL-USER::S5) 200000.0)
  )

(CPAD::USER-ACTIONS
   ((EQUATIONS-MENTIONING ((CL-USER::MASS-FLOW CL-USER::S7) CL-USER::NVALUE :UNKNOWN)) .
3136218056)
   ((ASSIGN (CL-USER::DRYNESS CL-USER::S7) 1) . 3136218011)
   ((RETRACT-ASN (CL-USER::ISO-PARAMETRIC CL-USER::SPL1)) . 3136217999)
   ((NVALUE-NEEDS ((CL-USER::POWER-IN :CYCLE) CL-USER::NVALUE :UNKNOWN)) . 3136217977)
   ((NVALUE-NEEDS ((CL-USER::NET-POWER :CYCLE) CL-USER::NVALUE :UNKNOWN)) . 3136217962)
   ((EQUATIONS-MENTIONING ((CL-USER::ETA-THERMAL :CYCLE) CL-USER::NVALUE :UNKNOWN)) . 3136217948)
   ((UNASSIGN (CL-USER::FLOW-FRACTION CL-USER::S5) 1.0) . 3136217888)
   ((UNASSIGN (CL-USER::MASS-FLOW CL-USER::S6) 0.134527) . 3136217822)
   ((INVOKE-ASN (CL-USER::ISOBARIC CL-USER::MXR1)) . 3136217810)
   ((CL-USER::NVALUE-NEEDS ((CL-USER::FLOW-SPLIT CL-USER::SPL1) CL-USER::NVALUE :UNKNOWN)) .
3133634462)
   ((CL-USER::EQUATIONS-MENTIONING ((CL-USER::FLOW-SPLIT CL-USER::SPL1) CL-USER::NVALUE
```

```
:UNKNOWN)) . 3133634424)
   ((CL-USER::EQUATIONS-MENTIONING ((CL-USER::SPEC-H CL-USER::S11) CL-USER::NVALUE 504800.0)) .
3133634366)
   ((CL-USER::EQUATIONS-MENTIONING ((CL-USER::FLOW-SPLIT CL-USER::SPL1) CL-USER::NVALUE
:UNKNOWN)) . 3133633140)
   ((CL-USER::EXPLAIN-CONSEQUENCES (CL-USER::EQUATION (:= (CL-USER::FLOW-FRACTION CL-USER::S7) (/
(CL-USER::REFERENCE-FLOW-FRACTION CL-USER::S7) (CL-USER::MAX-REFERENCE-FLOW :CYCLE))))) .
3133633099)
   ((CL-USER::EQUATIONS-MENTIONING ((CL-USER::FLOW-FRACTION CL-USER::S7) CL-USER::NVALUE
0.865473)) . 3133633096)
   ((WHY (CL-USER::EQUATION (:= (CL-USER::FLOW-FRACTION CL-USER::S7) (/ (CL-USER::REFERENCE-FLOW-
FRACTION CL-USER::S7) (CL-USER::MAX-REFERENCE-FLOW :CYCLE))))) . 3133633091)
   ((CL-USER::EQUATIONS-MENTIONING ((CL-USER::FLOW-FRACTION CL-USER::S7) CL-USER::NVALUE
0.865473)) . 3133633076)
   ((CL-USER::EQUATIONS-MENTIONING ((CL-USER::MASS-FLOW CL-USER::S7) CL-USER::NVALUE 0.865473)) .
3133633062)
   ((CL-USER::ASSIGN (CL-USER::MASS-FLOW CL-USER::S6) 0.134527) . 3133630645)
   ((CL-USER::EQUATIONS-MENTIONING ((CL-USER::SPEC-H CL-USER::S11) CL-USER::NVALUE 504800.0)) .
3133629954)
   ((CL-USER::EXPLAIN-CONSEQUENCES (CL-USER::EQUATION (:= (CL-USER::SPEC-H CL-USER::S11) (/ (CL-
USER::H-DOT CL-USER::S11) (CL-USER::MASS-FLOW CL-USER::S11))))) . 3133629946)
   ((CL-USER::EQUATIONS-MENTIONING ((CL-USER::H-DOT CL-USER::S11) CL-USER::NVALUE 504800.0)) .
3133629936)
   ((CL-USER::EQUATIONS-MENTIONING ((CL-USER::FLOW-SPLIT CL-USER::SPL1) CL-USER::NVALUE
:UNKNOWN)) . 3133629877)
   ((CL-USER::ASSIGN (CL-USER::FLOW-FRACTION CL-USER::S5) 1) . 3133629683)
   ((CL-USER::ASSIGN (CL-USER::P CL-USER::S5) 200000.0) . 3091141870)
   ((CL-USER::UNASSIGN (CL-USER::P CL-USER::S5) 800000.0) . 3091141859)
   ((CL-USER::ASSIGN (CL-USER::MASS-FLOW CL-USER::S1) 1) . 3091141304)
   ((CL-USER::ASSIGN (CL-USER::DRYNESS CL-USER::S11) 0) . 3091141288)
   ((CL-USER::INVOKE-ASN (SATURATED CL-USER::S11)) . 3091141278)
   ((CL-USER::RETRACT-ASN (SATURATED CL-USER::S12)) . 3091141270)
   ((CL-USER::INVOKE-ASN (SATURATED CL-USER::S12)) . 3091141263)
   ((CL-USER::INVOKE-ASN (== (CL-USER::P CL-USER::S10) (CL-USER::P CL-USER::S5))) . 3091141241)
   ((CL-USER::ASSIGN (CL-USER::DRYNESS CL-USER::S9) 0) . 3091141225)
   ((CL-USER::INVOKE-ASN (SATURATED CL-USER::S9)) . 3091141219)
   ((CL-USER::ASSIGN (CL-USER::P CL-USER::S8) 10000.0) . 3091141207)
   ((CL-USER::ASSIGN (CL-USER::P CL-USER::S5) 800000.0) . 3091141187)
   ((CL-USER::INVOKE-ASN (CL-USER::ISENTROPIC CL-USER::PMP2)) . 3091141172)
   ((CL-USER::INVOKE-ASN (CL-USER::ADIABATIC CL-USER::PMP2)) . 3091141167)
   ((CL-USER::INVOKE-ASN (CL-USER::ADIABATIC CL-USER::TUR2)) . 3091141158)
   ((CL-USER::INVOKE-ASN (CL-USER::ISENTROPIC CL-USER::TUR2)) . 3091141154)
   ((CL-USER::INVOKE-ASN (CL-USER::ISO-PARAMETRIC CL-USER::SPL1)) . 3091141143)
   ((CL-USER::ASSIGN (CL-USER::P CL-USER::S1) 5000000.0) . 3091141121)
   ((CL-USER::ASSIGN (T CL-USER::S1) 673.15) . 3091141120)
   ((CL-USER::UNASSIGN (CL-USER::REFERENCE-FLOW-FRACTION CL-USER::S1) 1.0) . 3091141119)
   ((CL-USER::UNASSIGN (CL-USER::FLOW-SPLIT CL-USER::SPL1) 0.98) . 3091141117)
   ((CL-USER::ASSIGN (CL-USER::FLOW-SPLIT CL-USER::SPL1) 0.98) . 3091141116)
   ((CL-USER::UNASSIGN (CL-USER::FLOW-SPLIT CL-USER::SPL1) 0.02) . 3091141115)
   ((CL-USER::ASSIGN (CL-USER::FLOW-SPLIT CL-USER::SPL1) 0.02) . 3091141114)
   ((CL-USER::UNASSIGN (CL-USER::FLOW-SPLIT CL-USER::SPL1) 0.5) . 3091141113)
   ((CL-USER::ASSIGN (CL-USER::FLOW-SPLIT CL-USER::SPL1) 0.5) . 3091141112)
   ((CL-USER::ASSIGN (CL-USER::REFERENCE-FLOW-FRACTION CL-USER::S1) 1.0) . 3091141109)
   ((CL-USER::UNASSIGN (CL-USER::FLOW-SPLIT CL-USER::SPL1) 0.5) . 3091141108)
   ((CL-USER::UNASSIGN (CL-USER::REFERENCE-FLOW-FRACTION CL-USER::S1) 1.0) . 3091141107)
   ((CL-USER::ASSIGN (CL-USER::REFERENCE-FLOW-FRACTION CL-USER::S1) 1.0) . 3091141104)
   ((CL-USER::ASSIGN (CL-USER::FLOW-SPLIT CL-USER::SPL1) 0.5) . 3091141103)
   ((CL-USER::CONNECTED CL-USER::PMP1 CL-USER::HTR1) . 3091141058)
   ((CL-USER::CONNECTED CL-USER::MXR1 CL-USER::PMP1) . 3091141054)
   ((CL-USER::CONNECTED CL-USER::PMP2 CL-USER::MXR1) . 3091141041)
   ((CL-USER::CONNECTED CL-USER::CLR1 CL-USER::PMP2) . 3091141028)
   ((CL-USER::CONNECTED CL-USER::TUR2 CL-USER::CLR1) . 3091141014)
   ((CL-USER::CONNECTED CL-USER::SPL1 CL-USER::TUR2) . 3091141001)
   ((CL-USER::CONNECTED CL-USER::SPL1 CL-USER::MXR1) . 3091140983)
   ((CL-USER::CONNECTED CL-USER::TUR1 CL-USER::SPL1) . 3091140960)
   ((CL-USER::ADD-DEVICE PUMP "PMP2" CL-USER::PMP2) . 3091140931)
   ((CL-USER::ADD-DEVICE TURBINE "TUR2" CL-USER::TUR2) . 3091140929)
   ((CL-USER::ADD-DEVICE MIXER "MXR1" CL-USER::MXR1) . 3091140925)
   ((CL-USER::ADD-DEVICE SPLITTER "SPL1" CL-USER::SPL1) . 3091140922)
   ((CL-USER::DELETED CL-USER::STUFF CL-USER::S4) . 3091140909)
   ((CL-USER::DELETED CL-USER::STUFF CL-USER::S3) . 3091140907)
   ((CL-USER::DELETED CL-USER::STUFF CL-USER::S2) . 3091140906)
   ((CL-USER::UNASSIGN (CL-USER::DRYNESS CL-USER::S3) 0) . 3091140884)
   ((CL-USER::UNASSIGN (CL-USER::P CL-USER::S2) 10000.0) . 3091140884)
   ((CL-USER::UNASSIGN (CL-USER::P CL-USER::S1) 5000000.0) . 3091140883)
   ((CL-USER::UNASSIGN (T CL-USER::S1) 673.15) . 3091140881)
   ((CL-USER::INVOKE-ASN (CL-USER::ISOBARIC CL-USER::HTR1)) . 3091138876)
   ((CL-USER::INVOKE-ASN (CL-USER::ADIABATIC CL-USER::PMP1)) . 3091138868)
   ((CL-USER::INVOKE-ASN (CL-USER::ISENTROPIC CL-USER::PMP1)) . 3091138863)
   ((CL-USER::ASSIGN (CL-USER::DRYNESS CL-USER::S3) 0) . 3091138859)
   ((CL-USER::INVOKE-ASN (SATURATED CL-USER::S3)) . 3091138851)
```

```
  ((CL-USER::INVOKE-ASN (CL-USER::ISOBARIC CL-USER::CLR1)) . 3091138843)
  ((CL-USER::ASSIGN (CL-USER::P CL-USER::S2) 10000.0) . 3091138835)
  ((CL-USER::INVOKE-ASN (CL-USER::ADIABATIC CL-USER::TUR1)) . 3091138826)
  ((CL-USER::INVOKE-ASN (CL-USER::ISENTROPIC CL-USER::TUR1)) . 3091138823)
  ((CL-USER::ASSIGN (CL-USER::P CL-USER::S1) 5000000.0) . 3091138814)
  ((CL-USER::ASSIGN (T CL-USER::S1) 673.15) . 3091138808)
  ((CL-USER::INVOKE-ASN (SUBSTANCE-OF CL-USER::S1 WATER)) . 3091138802)
  ((CL-USER::CONNECTED CL-USER::PMP1 CL-USER::HTR1) . 3091138719)
  ((CL-USER::CONNECTED CL-USER::CLR1 CL-USER::PMP1) . 3091138716)
  ((CL-USER::CONNECTED CL-USER::TUR1 CL-USER::CLR1) . 3091138712)
  ((CL-USER::CONNECTED CL-USER::HTR1 CL-USER::TUR1) . 3091138709)
  ((CL-USER::ADD-DEVICE PUMP "PMP1" CL-USER::PMP1) . 3091138683)
  ((CL-USER::ADD-DEVICE COOLER "CLR1" CL-USER::CLR1) . 3091138680)
  ((CL-USER::ADD-DEVICE TURBINE "TUR1" CL-USER::TUR1) . 3091138677)
  ((CL-USER::ADD-DEVICE HEATER "HTR1" CL-USER::HTR1) . 3091138671)
  ((CL-USER::INVOKE-ASN (CL-USER::HEAT-ENGINE :CYCLE)) . 3091138663)
  ((CL-USER::INVOKE-ASN (:NOT (CL-USER::CONTROL-FLAG :ISENTROPIC-MEANS-IDEAL))) . 3091138656)
  ((CL-USER::INVOKE-ASN (:NOT (CL-USER::CONTROL-FLAG :CONSIDER-VELOCITY))) . 3091138655)
  ((CL-USER::INVOKE-ASN (:NOT (CL-USER::CONTROL-FLAG :CONSIDER-ECONOMICS))) . 3091138655)
  ((CL-USER::INVOKE-ASN (:NOT (CL-USER::CONTROL-FLAG :USE-AIR-STANDARD))) . 3091138654)
  ((CL-USER::INVOKE-ASN (:NOT (CL-USER::CONTROL-FLAG :USE-TENV))) . 3091138654)
  ((CL-USER::INVOKE-ASN (:NOT (CL-USER::CONTROL-FLAG :PROPAGATE-SENSITIVITY))) . 3091138654))

(CPAD::PROBLEM-DESC
  "NIL NIL NIL NIL NIL NIL NIL NIL")
```
